# The convergent and divergent patterns in brain perfusion between Alzheimer's disease and Parkinson's disease with dementia: An ASL MRI study

**DOI:** 10.3389/fnins.2022.892374

**Published:** 2022-11-03

**Authors:** Hongri Chen, Yao Xu, Lanlan Chen, Songan Shang, Xianfu Luo, Xin Wang, Wei Xia, Hongying Zhang

**Affiliations:** ^1^Department of Radiology, Clinical Medical College, Yangzhou University, Yangzhou, China; ^2^Department of Radiology, Weihai Maternal and Child Health Care Hospital, Weihai, China; ^3^Department of Neurology, Clinical Medical College, Yangzhou University, Yangzhou, China; ^4^Department of Rehabilitation, Clinical Medical College, Yangzhou University, Yangzhou, China

**Keywords:** Parkinson's disease, Alzheimer's disease, arterial spin labeling, perfusion, dementia

## Abstract

**Background:**

Aberrant brain blood perfusion changes have been found to play an important role in the progress of Alzheimer's disease (AD) and Parkinson's disease with dementia (PDD). However, the convergent and divergent patterns in brain perfusion between two dementias remain poorly documented.

**Objective:**

To explore the impaired brain perfusion pattern and investigate their overlaps and differences between AD and PDD using normalized cerebral blood flow (CBF).

**Methods:**

The regional perfusion in patients with AD and PDD as well as healthy control (HC) subjects were explored using the three-dimensional arterial spin labeling. The normalized CBF values were compared across the three groups and further explored the potential linkages to clinical assessments.

**Results:**

In total, 24 patients with AD, 26 patients with PDD, and 35 HC subjects were enrolled. Relative to the HC group, both the AD group and the PDD group showed reduced normalized CBF mainly in regions of the temporal and frontal gyrus, whereas preserved perfusion presented in the sensorimotor cortex and basal ganglia area. Compared with the AD group, the PDD group showed decreased perfusion in the right putamen and right supplementary motor area (SMA), while preserved perfusion in the right inferior parietal lobule (IPL) and right precuneus. In the AD group, significant correlations were observed between the normalized CBF values in the right IPL and scores of global cognitive function (*P* = 0.033, ρ = 0.442), between the normalized CBF values in the right precuneus and the scores of memory function (*P* = 0.049,ρ = 0.406). The normalized CBF in the right putamen was significantly linked to cores of motor symptoms (*P* = 0.017, ρ = 0.214) in the PDD group.

**Conclusion:**

Our findings suggested convergent and divergent patterns of brain hemodynamic dysregulation between AD and PDD and contributed to a better understanding of the pathophysiological mechanisms.

## Introduction

Alzheimer's disease (AD) and Parkinson's disease with dementia (PDD) are the two pronounced phenotypes of dementia characterized by progressive cognitive dysfunction. Patients with AD suffer from prominent memory loss as well as various behavioral and psychiatric dysfunctions (Dubois et al., 2021). As the predominant non-motor symptom of Parkinson's disease (PD), which is known as an aging-related movement disorder, PDD is accompanied by heterogeneous impaired cognitive domains (Aarsland et al., 2005). Although tau and Aβ are the hallmarks of AD while α-synuclein is the pathological feature of PDD, the presence of copathologies has been reported in these two dementias as well as the commonalities in their phenotypic clinical presentations (Irwin et al., 2013; Rodriguez-Vieitez and Nielsen, 2019), suggesting that there could exist overlapped underlying mechanisms.

Magnetic resonance imaging (MRI) structural measurements have been utilized by several studies and general findings suggested greater medial temporal lobe atrophy involvement in AD vs. PDD (Tam et al., 2005). However, the mechanism modulating the evolution of dementia should be further investigated from the aspect of brain perfusion, given that cerebral blood flow (CBF) has an important effect on both the etiology and progression of AD and PDD (Zhang et al., 2017; Domingues et al., 2021). Currently, arterial spin labeling (ASL) has been widely employed in quantitative measurement of CBF *via* magnetically labeling blood water spins without the need for contrast agent injection (Alsop et al., 2015), which is increasingly implemented in clinical and scientific research as an alternative for brain perfusion measurement (Cha et al., 2013).

Although aberrant perfusion has been observed in the frontal and posterior brain regions with relevance to cognitive decline in both AD and PDD (Schonberger et al., 2015; Duan et al., 2020), the regional CBF alterations underlying pathology might be overlooked (Aslan and Lu, 2010) due to the large intersubject variations in absolute CBF value among individuals. The normalized CBF has thus been verified by emerging literature, which has been proven to be more sensitive than absolute CBF (Aslan and Lu, 2010; Melzer et al., 2011). Even though, the convergent and divergent perfusion alterations between AD and PDD still remain unclear by means of normalized CBF. In this study, we aimed to explore commonalities and differences in perfusion patterns of the normalized CBF across the whole brain incorporated in both dementia groups. The potential relationships between the regional normalized CBF values and clinical characteristics were further investigated for the understanding of the underlying mechanisms.

## Materials and methods

### Participants

Patients with dementia were consecutively recruited from the Department of Neurology in our hospital from July 2019 to November 2020. A total of 85 right-handed participants were finally recruited for this study, comprising 24 patients (17 males and 7 females, mean age: 68.95 ± 6.97 years) with AD, 26 patients with PDD (18 males and 8 females, mean age: 68.50 ± 7.01 years), and 35 healthy control (HC) subjects (18 males and 17 females, mean age: 68.60 ± 3.98 years) matched for age, sex, handedness, and years of education. All the enrolled participants were clinically evaluated by two experienced neurologists. PDD was diagnosed according to the International Parkinson's and Movement Disorder Society (MDS) criteria (Emre et al., 2007). Patients with AD met the core clinical National Institute on Aging and Alzheimer's Association (NIA-AA) criteria (McKhann et al., 2011). All patients were instructed to refrain from taking medication for at least 12 h before arrival at the MRI facility. All participants provided written informed consent, and the study was approved by the Medical Research Ethical Committee in our Hospital (No. J-2014066).

Exclusion criteria were as follows: (a) other types of dementia, such as dementia with Lewy bodies (McKeith et al., 2017), frontotemporal dementia (Rascovsky et al., 2011), and vascular dementia (Roman et al., 1993); (b) multiple system atrophy, progressive supranuclear palsy, and other Parkinson's superimposed syndromes; (c) severe motion artifacts during MRI, severe head tremor, deafness, blindness, and coma; (d) history of other neurological or psychiatric diseases, such as depression, epilepsy, schizophrenia, encephalitis, and traumatic brain injury; (e) metabolic diseases, long-term alcoholism, and drug addicts; (f) additional brain lesions detected on structural MR imaging, such as stroke, brain tumor, and Fazekas scale of white matter hyperintensities > 2; and (g) contraindications for MRI, such as claustrophobia, fixed dentures or other metal implants, pregnancy. Additionally, HC subjects were excluded if they had complaints of cognitive impairment or first-degree family members with Parkinsonism or dementia.

The Movement Disorder Society-Unified Parkinson's Disease Rating Scale (MDS-UPDRS) Part III and the Hoehn & Yahr (H&Y) stages were used to assess motor symptoms of PDD and the stage of the disease. The Mini-Mental State Examination (MMSE) and Montreal Cognitive Assessment (MoCA) were used to assess global cognitive functions in the three groups, and the severity of dementia was measured by the clinical dementia rating (CDR) scale. Moreover, the logical memory-immediate recall (LMI) test, the digit span test (DST), the symbol digit modalities test (SDMT), the clock drawing test (CDT), and the Boston naming test (BNT) were used to assess memory, attention, executive, visual space, and language function, respectively. The demographic and clinical data of all participants are shown in Table 1.

**Table 1:** Demographic and clinical data of all subjects.

	**AD (*n* = 24)**	**PDD (*n* = 26)**	**HC (*n* = 35)**	***P-*value**
Age (years)	68.95 ± 6.97	68.50 ± 7.01	68.60 ± 3.98	0.96^a^
Sex (male/female)	17/7	18/8	18/17	0.17^b^
Education (years)	9.00 (2.00,6.00)	6.00 (5.50,10.50)	9.00 (2.50,6.00)	0.70^a^
Duration (years)	5.91 ± 3.26	6.12 ± 4.57	-	0.98^c^
MMSE	15.71 ± 3.93	14.12 ± 4.11	28.75 ± 1.49	0.36^a^
MoCA	14.46 ± 4.26	14.15 ± 3.48	27.28 ± 1.17	0.48^a^
CDR	1.83 ± 0.75	1.92 ± 0.73	-	0.83^c^
LMI	4.83 ± 2.59	13.23 ± 3.95	24.41 ± 4.56	**< 0.001** ^**a**^
DST	4.88 ± 2.09	4.81 ± 1.41	7.00 ± 1.05	0.83^a^
SDMT	36.25 ± 8.49	30.38 ± 6.67	43.12 ± 6.88	**0.01** ^a^
CDT	3.08 ± 0.70	1.50 ± 0.57	8.91 ± 4.46	**0.00** ^a^
BNT	19.21 ± 4.75	18.50 ± 4.32	26.4 ± 3.2	0.38^a^
UPDRS-III	-	34.72 ± 6.50	-	-
H andY	-	2.50 (2.00, 3.00)	-	-

a The P value was obtained by ANOVA or Kruskal–Wallis test across three groups.

b The P value was obtained by chi-square tests across three groups.

c The P value was obtained by two-sample t-tests between the AD and PDD groups. AD, Alzheimer's disease; PDD, Parkinson's disease with dementia; HC, healthy control; MMSE, Mini-Mental State Examination; MoCA, Montreal Cognitive Assessment; CDR, clinical dementia rating; LMI, logical memory-immediate recall; DST, digit span test; SDMT, symbol digit modalities test; CDT, clock drawing test; BNT, Boston naming test; UPDRS, Unified Parkinson's Disease Rating Scale; HandY, Hoehn and Yahr. Bold values indicates statistically significant differences with *P* < 0.05.

### MRI data acquisition

All MRI data were acquired using a 3.0 Tesla scanner (Discovery MR750, GE, Milwaukee, USA) with an 8-channel phased-array head coil. Routine structural MR imaging was performed before ASL to confirm whether there were other structural abnormalities. The subsequent imaging protocol included three-dimensional fast-spin-echo (3D-FSE) ASL with spiral readout. The protocol parameters were as follows: repetition time (TR) = 4,844 ms, echo time (TE) = 5.1 ms, postlabeling delay (PLD) = 2,025 ms, field of view (FOV) = 240 × 240 mm, matrix size = 64 × 64, and number of excitations = 3. The scanning time was 4 min 41 s. High-resolution sagittal three-dimensional T1-weighted brain volume imaging of the whole brain was collected with the following parameters: TR = 12 ms, TE = 5.1 ms, TI = 450 ms, FOV = 256 × 256 mm, matrix size = 256 × 256, 1 mm slice thickness, and 0 mm slice spacing. The scanning time was 4 min 02 s.

### MRI data processing and analysis

The CBF maps derived from ASL were extracted based on the Functool software (version 10.4.04, GE Healthcare), and data preprocessing and analyses were carried out using custom scripts in MATLAB (R2013b, MathWorks, MA) and Statistical Parametric Mapping software (SPM12, http://www.fil.ion.ucl.ac.uk/spm). ASL images were first corrected for motion. According to the motion parameters provided by SPM, participants with translations and rotations higher than 2 mm and 2° were removed from the analysis. Each participant's CBF image was coregistered to their structural images, and individual structural images were normalized to Montreal Neurological Institute (MNI) space; spatial transforms were concatenated to bring the CBF image to the MNI template, with resampling to a 2 × 2 × 2 mm voxel size. Normalized CBF was calculated by subtracting the global mean and dividing by the standard deviation (SD) and then smoothed with an 8 mm full width at half maximum (FWHM) Gaussian kernel. The relatively increased perfusion between groups is interpreted as preserved perfusion relative to the global mean.

We also calculated the gray matter (GM) volume (GMV) as a covariate for statistical analysis to regress the brain tissue atrophy factor. After the initial affinity of the segmented GM map in the T1 images was registered to MNI space, the GM concentration graph was non-linearly deformed by exponential Lie algebra, and the resampling voxel size was 1.5 × 1.5 × 1.5 mm. Smoothing was performed with an 8 mm FWHM Gaussian kernel on the GMV image.

### Statistical analyses

For the demographic analysis, sex data were analyzed with a chi-square test. Normality of continuous variable distributions was tested with the Kolmogorov–Smirnov test. If the data were normally distributed, they were expressed as ($\bar{x}$ ± s), and a *t*-test or analysis of variance (ANOVA) was used for comparisons between groups. For non-normally distributed data, expressed as the median and interquartile range [M (*P25, P75*)], a non-parametric Kruskal–Wallis test was used. All data were analyzed using the SPSS 25.0 software (SPSS, Chicago, IL, USA) and *P* < 0.05 was considered to be significant.

Imaging data were analyzed using SPM12. Analysis of covariance (ANCOVA) was conducted on the absolute and normalized CBF maps of AD, PDD, and HC subjects with age, sex, education level, and GMV as covariates, followed by a *post-hoc* two-sample *t*-test. Then, two-sample *t*-tests were performed within the mask showing conspicuous differences acquired from ANCOVA analysis. The statistical analyses were identified using a significance threshold of 0.05 after correction for multiple comparisons at the cluster level using the familywise error (FWE), with a cluster-generating threshold of *P* < 0.001.

We calculated the correlations between normalized CBF values and scores of clinical assessments. Brain regions with significant differences between dementia groups were saved as regions of interest (ROIs) and their normalized CBF values were then extracted. The significance threshold of correlation analysis was set at *P* < 0.05 using the Spearman rank correlation method, taking age, sex, educational level, and GMV as covariates. Receiver operating characteristic (ROC) analysis was constructed on the MedCalc software (version 15.6, MedCalc Software, Ostend, Belgium) to ascertain the recognition capacity efficacy of regional CBF to discriminate patients with AD from patients with PDD.

## Results

### Demographic and clinical characteristics

Demographic and clinical data are listed in Table 1. Age, sex, and education level did not differ among the three groups (all *P* > 0.05). Both the AD and PDD groups showed significantly lower MMSE and MoCA scores than the HC group (*P* < 0.05), and no significant difference was observed between the dementia groups (*P* > 0.05). In addition, the memory scores (logical memory-immediate recall scores, *P* < 0.001) in the AD group were significantly lower than those in the PDD group, while patients with PDD had lower executive scores (SDMT, *P* = 0.01) and visual space scores (CDT, *P* < 0.001) than patients with AD.

### ANCOVA analysis of CBF

**Figure 1:**
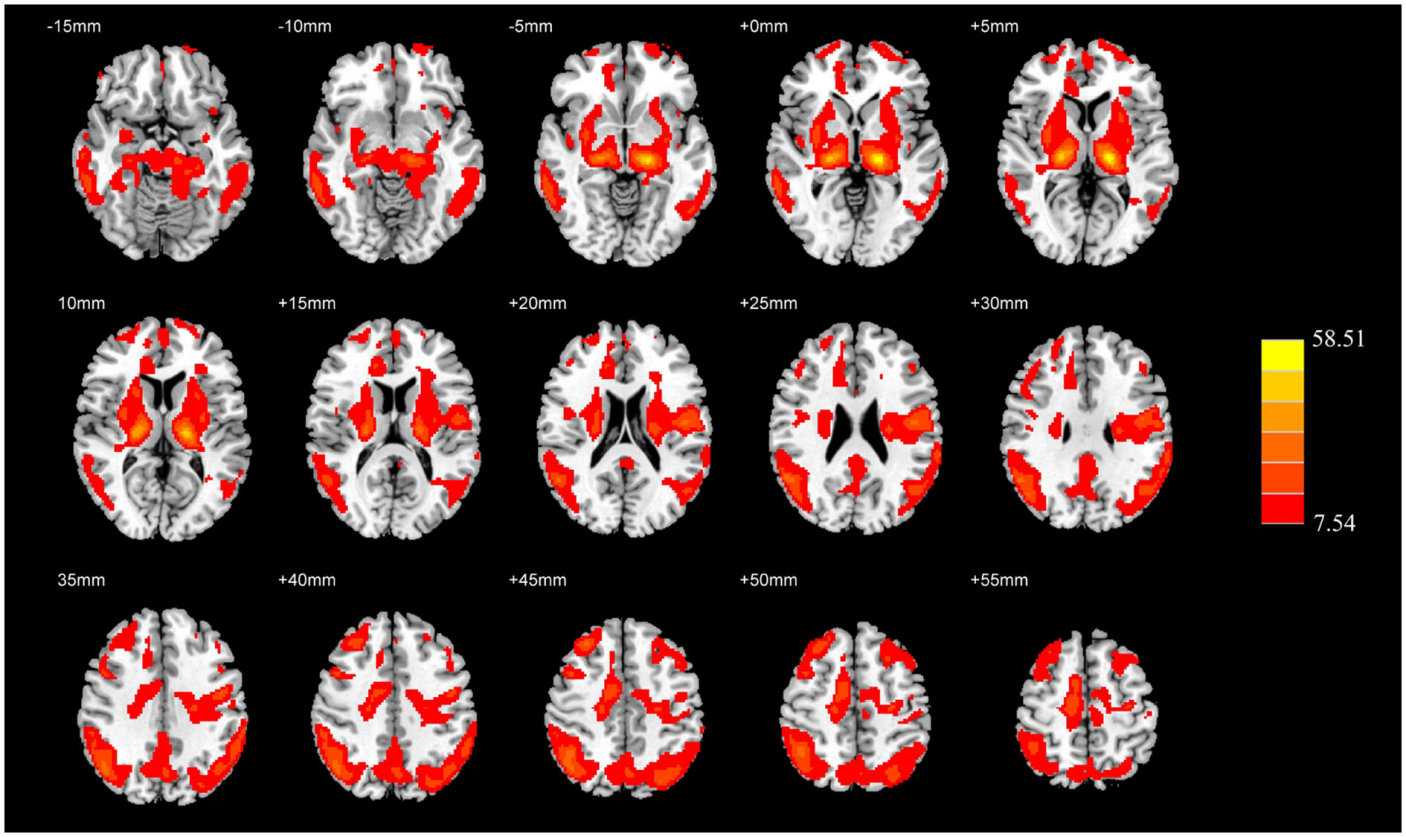
ANCOVA analysis of the normalized CBF among the HC, AD, and PDD groups. Significantly different value among groups is shown with warm color (*P*_FWE_ < 0.05). ANCOVA, Analysis of covariance; CBF, cerebral blood flow; HC, healthy control; AD, Alzheimer's disease; PDD, Parkinson's disease with dementia; FWE, familywise error.

The absolute CBF showed no significant differences among the three groups after adjusting for age, sex, education level, and GMV (*P*_FWE_ > 0.05). The analysis after covariate adjustment showed that the normalized CBF maps allowed the identification of the regions with significant differences among the HC, AD, and PDD groups. These regions include the widespread cortical regions of the frontal, parietal, and temporal cortices and subcortical structures of the basal ganglia (*P*_FWE_ < 0.05) (Figure 1). Therefore, the normalized CBF data were entered into the subsequent analysis.

### Group-level differences in normalized CBF

**Figure 2:**
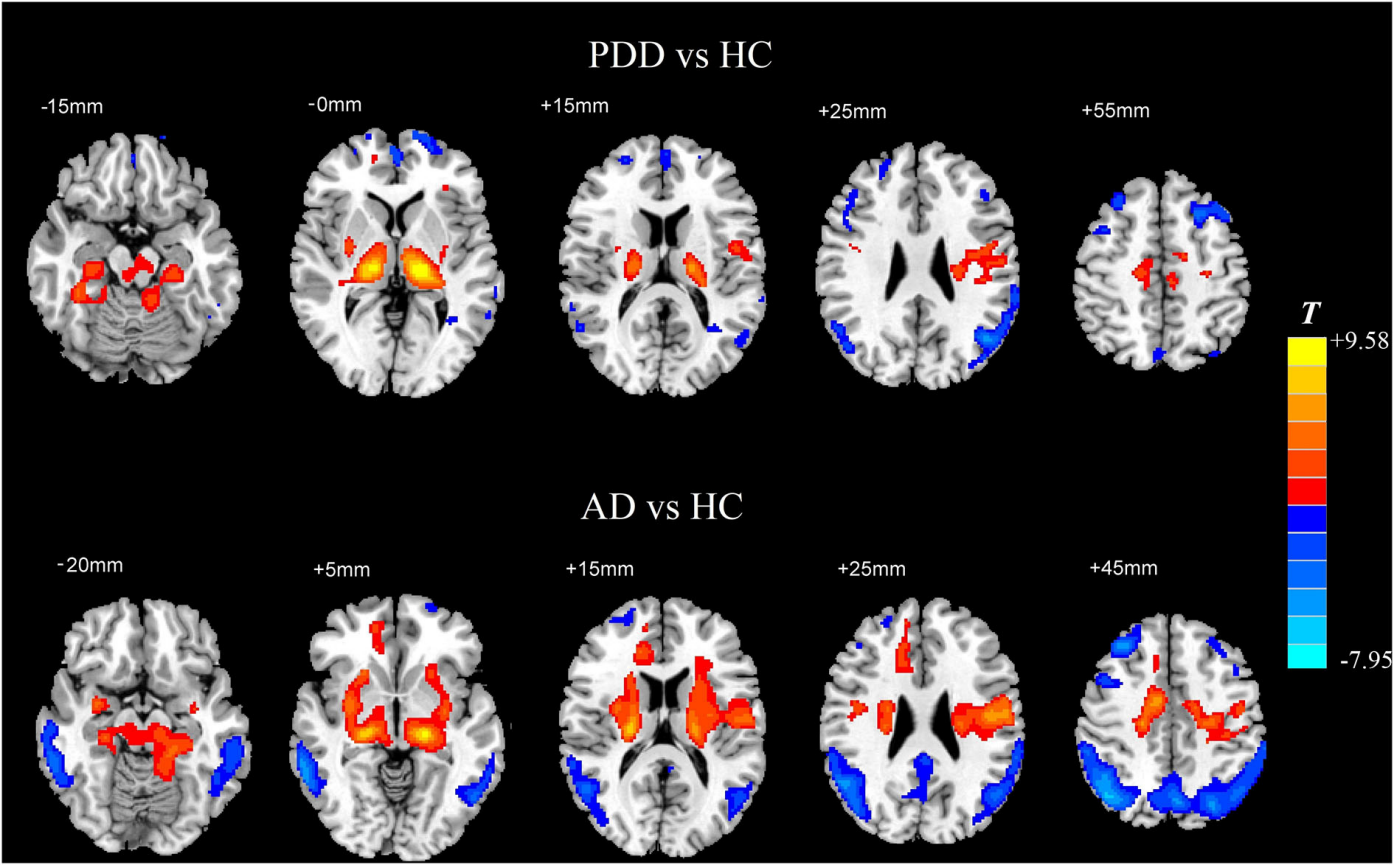
Two-sample *t*-test of normalized CBF between each dementia group and the HC group, respectively. Significantly increased value in the group is shown with warm color, while significantly decreased value in the group is shown with cold color (*P*_FWE_ < 0.05). CBF, cerebral blood flow; HC, healthy control; AD, Alzheimer's disease; PDD, Parkinson's disease with dementia; FWE, familywise error.

Compared with the HC group, the AD group showed reduced normalized CBF mainly in regions of bilateral middle temporal gyrus and bilateral middle frontal gyrus (MFG), whereas preserved perfusion presented in the left postcentral gyrus and right putamen (two-sample *t*-test, *P*_FWE_ < 0.05) (details in Table 2; Figure 2).

**Table 2:** Descriptions of brain regions with significant perfusion differences by *post-hoc* analysis between the AD and HC groups.

**Brain regions (AAL)**	**Peak MNI coordinates**			**Peak *T* value**	**Cluster size (voxels)**
	**X**	**Y**	**Z**		
**AD < HC**					
Right middle temporal gyrus	42	−68	44	−7.306	6,286
Left middle temporal gyrus	−4	−72	40	−6.961	7,731
Right middle frontal gyrus	32	32	44	−6.839	2,033
Left middle frontal gyrus	−24	22	52	−4.771	601
**AD > HC**					
Left postcentral gyrus	22	−22	2	9.472	17,599
Right putamen	10	−4	46	6.337	2,718

AD, Alzheimer's disease; HC, healthy control; AAL, Anatomical automatic labeling; MNI, Montreal Neurological Institute.

In comparison with the HC group, the PDD group showed reduced normalized CBF mainly in regions of bilateral MFG, bilateral angular gyrus, left inferior temporal gyrus, and left superior frontal gyrus, whereas preserved perfusion presented in the left thalamus and right supplementary motor area (SMA) (two-sample *t*-test, *P*_FWE_ < 0.05) (details in Table 3; Figure 2).

**Table 3:** Descriptions of brain regions with significant perfusion differences by *post-hoc* analysis between the PDD and HC groups.

**Brain regions (AAL)**	**Peak MNI coordinates**			**Peak *T* value**	**Cluster size (voxels)**
	**X**	**Y**	**Z**		
**PDD < HC**					
Left inferior temporal gyrus	−50	−62	−6	−4.034	251
Left superior frontal gyrus	−22	66	−12	−5.381	309
Right middle frontal gyrus	30	32	46	−5.614	259
Left angular gyrus	−44	−64	28	−5.503	1,410
Right angular gyrus	46	−68	38	−4.852	352
Left middle frontal gyrus	−24	22	50	−5.407	490
**PDD > HC**					
Left thalamus	−16	−22	6	8.689	9,087
Right supplementary motor area	−8	−24	66	4.818	873

PDD, Parkinson's disease with dementia; HC, healthy control; AAL, Anatomical automatic labeling; MNI, Montreal Neurological Institute.

**Figure 3:**
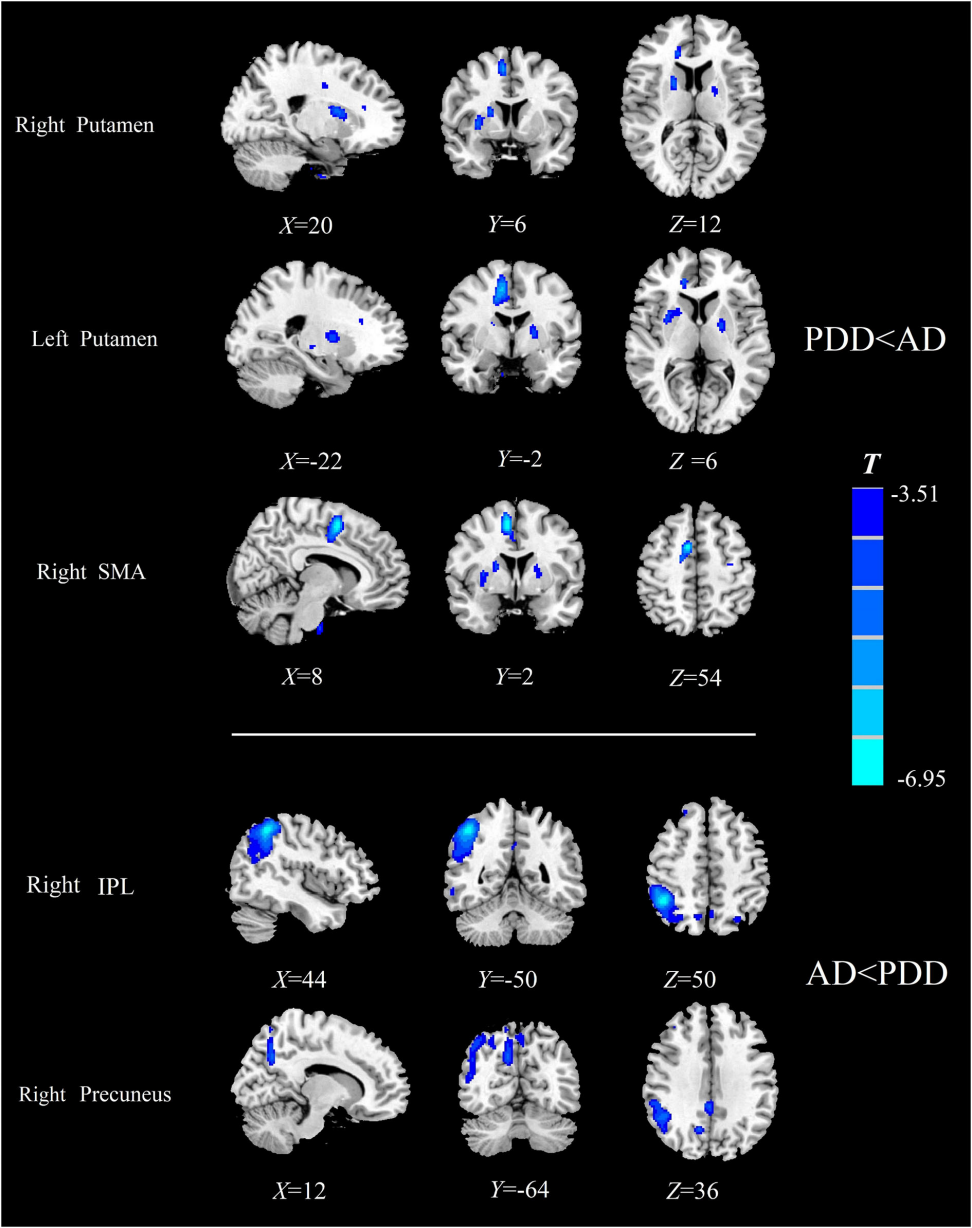
Comparison of the normalized CBF between patients with AD and patients with PDD. Significantly decreased value in the group is shown with cold color (*P*_FWE_ < 0.05). CBF, cerebral blood flow; AD, Alzheimer's disease; PDD, Parkinson's disease with dementia; FWE, familywise error; SMA, supplementary motor area; IPL, inferior parietal lobule.

Specially, we also found different perfusion distributions between dementia groups, although the abovementioned similar perfusion pattern was observed for both groups. When compared with patients with AD, patients with PDD showed lower perfusion in the bilateral putamen and right SMA, while higher normalized CBF in the right inferior parietal lobule (IPL) and right precuneus (*P*_FWE_ < 0.05) (details in Table 4; Figure 3).

**Table 4:** Descriptions of brain regions with normalized CBF differences by *post-hoc* analysis between the PDD and AD groups.

**Brain regions (AAL)**	**Peak MNI coordinates**			**Peak *T* value**	**Cluster size (voxels)**
	**X**	**Y**	**Z**		
**PDD < AD**					
Right putamen	20	6	12	−4.130	488
Left putamen	−22	−2	6	−3.922	382
Right supplementary motor area	8	2	54	−5.062	682
**AD < PDD**					
Right inferior parietal lobule	44	−50	50	−6.432	3,805
Right precuneus	12	−64	36	−4.578	488

AD, Alzheimer's disease; PDD, Parkinson's disease with dementia; AAL, Anatomical automatic labeling; MNI, Montreal Neurological Institute.

### Correlations between CBF and clinical assessments

**Figure 4:**
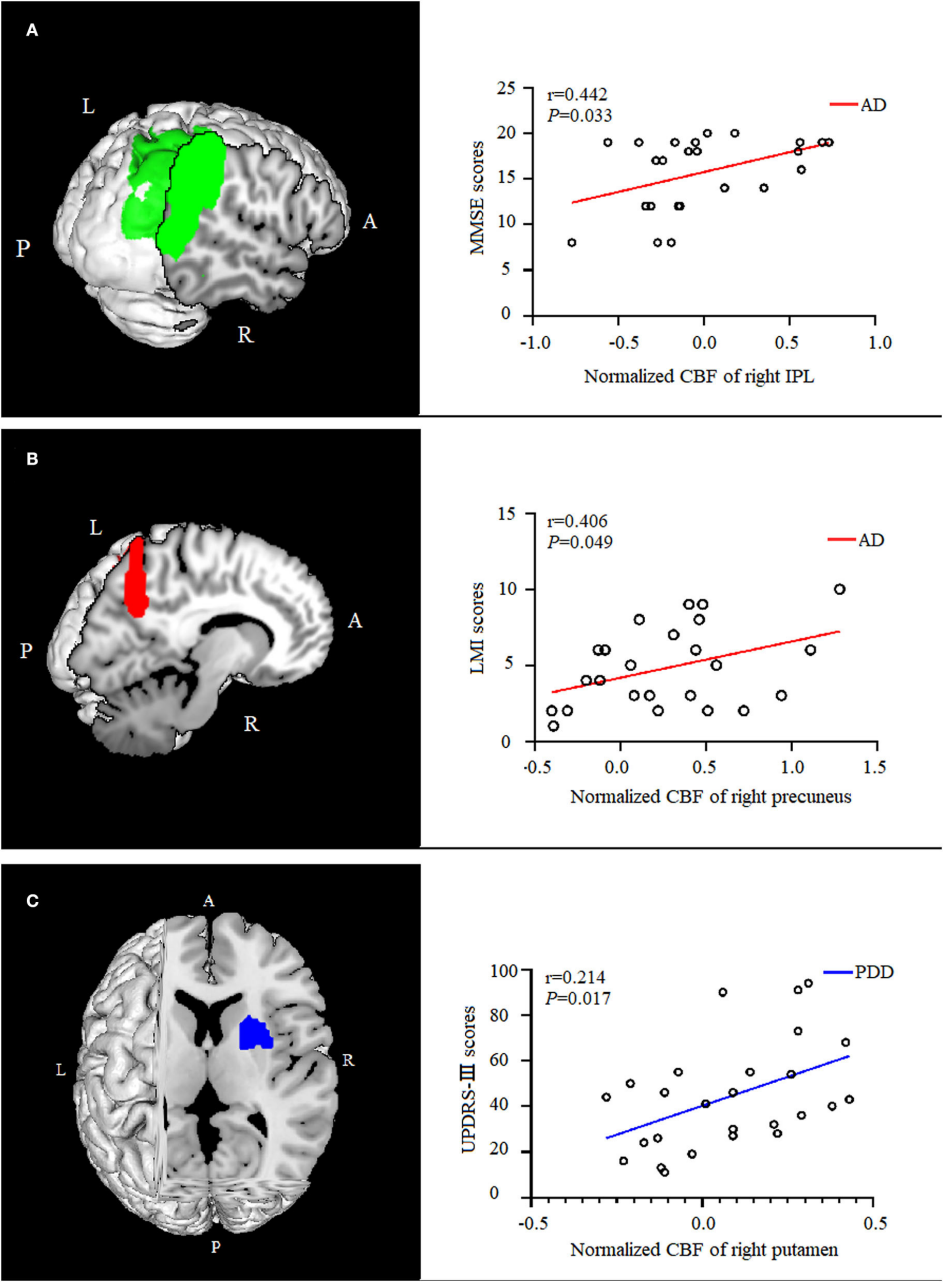
Correlation analysis between normalized CBF values and scores of clinical assessments. In the AD group, the normalized CBF of the right IPL were positively correlated with the MMSE scores [**(A)**, *P* = 0.033, ρ = 0.442], and the normalized CBF of were positively correlated with the right precuneus memory scores [**(B)**, *P* = 0.049,ρ = 0.406]. The normalized CBF of the right putamen showed a positive relationship with UPDRS-III scores in the PDD group [**(C)**, *P* = 0.017, ρ = 0.214]. CBF, cerebral blood flow; AD, Alzheimer's disease; MMSE, Mini-Mental State Examination; IPL, inferior parietal lobule; LMI, logical memory-immediate recall; UPDRS, Unified Parkinson's Disease Rating Scale; PDD, Parkinson's disease with dementia.

In the AD group, the normalized CBF values in the right IPL were positively correlated with (*P* = 0.033, ρ = 0.442) the MMSE scores, and the normalized CBF values in the right precuneus were positively correlated with (*P* = 0.049, ρ = 0.406) the LMI scores. The normalized CBF in the right putamen showed a positive relationship with (*P* = 0.017, ρ = 0.214) UPDRS-III scores in the PDD group (Figure 4).

### ROC analysis for dementia disease classification

**Figure 5:**
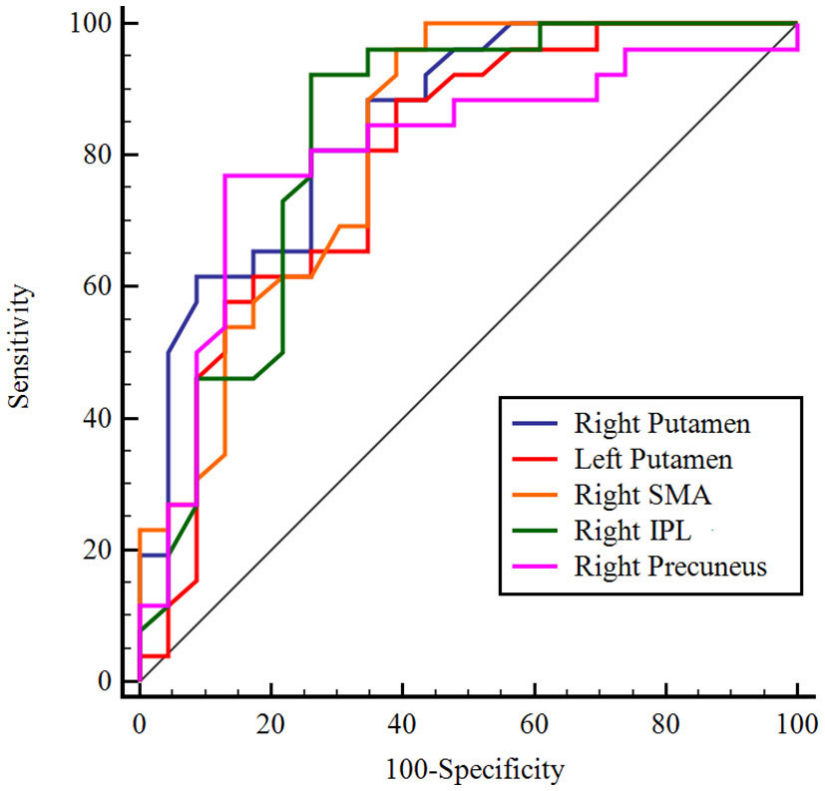
ROC analysis of normalized CBF in regions with significant differences to distinguish patients with AD from patients with PDD. The figure indicates that AUC was greatest for the right putamen. Different color lines represent different regions. ROC, receiver operating characteristic; CBF, cerebral blood flow; AD, Alzheimer's disease; PDD, Parkinson's disease with dementia; AUC, area under the curve; SMA, supplementary motor area; IPL, inferior parietal lobule.

Normalized CBF values were extracted from the five regions found in the above comparison between patients with AD and PDD and entered into ROC analysis. ROC curve analysis results showed that the normalized CBF values had sensitivity ranging from 81 to 96% and specificity ranging from 63 to 88%. The area under the curve (AUC) was greatest for the right putamen (AUC: 0.856, 95% CI: 0.728–0.939; cutoff value: −0.03, sensitivity: 81%, specificity: 75.0%; Figure 5).

## Discussion

In this study, we explored cerebral blood perfusion alterations in patients with AD and PDD using the CBF metric derived from ASL. Relative to HC subjects, patients with AD and PDD shared similar impaired perfusion patterns with altered normalized CBF. Moreover, several brain regions with different perfusion alterations were observed between two dementia groups, which were correlated with impairments in cognition (AD) or motor dysfunction (PDD). In accordance with the documented overlaps and differences between both dementia diseases (Irwin et al., 2012, 2013; Rodriguez-Vieitez and Nielsen, 2019; Bartels et al., 2020), our findings provided insights into the underlying mechanism from the perspective of cerebral hemodynamics in patients with AD and PDD.

ASL is a robust method of assessing microvascular perfusion by CBF metrics, thus indirectly reflecting neuronal activity. CBF is thought to be tightly regulated by the interconnected neurovascular system (Zlokovic, 2011). Hypoperfusion has been suggested to contribute to the deposition and aggregation of misfolded proteins, such as amyloid, tau, and α-synuclein, in neurodegenerative diseases (Bangen et al., 2017; d'Errico and Meyer-Luehmann, 2020). Relative to HC subjects, we observed hypoperfusion in the frontal and temporal cortex among AD and PDD participants, which has been proven to be related to the impairment of executive and memory functions (Yoshiura et al., 2009; Raji et al., 2010).

In addition, we identified increased CBF in subcortical structures and supplement motor area, in line with previous findings that described PD-related patterns and Alzheimer's disease-related patterns characterized by increased subcortical metabolism (Eidelberg et al., 1994; Ma et al., 2007; Mattis et al., 2016). The reason for the increased subcortical perfusion in PDD might be caused by the enhanced inhibitory activity of the indirect pathway in the basal ganglia (Wichmann, 2018). With the increased subcortical perfusion in AD, there is a compensatory hypothesis that the basal ganglia are excited by the fronto-striato-thalamic circuit to compensate for the impaired cortical regions (Alsop et al., 2008; Ding et al., 2014).

Traditionally, PDD is thought of as subcortical dementia, and AD is related to more damage to the cortices. In our findings, besides the common perfusion pattern, patients with AD and PDD showed distinct perfusion features when compared with each other. Compared with patients with AD, patients with PDD presented hypoperfusion mainly in bilateral putamen and right SMA. These regions belong to the striatal-thalamo-cortical (STC) circuit and play a critical role in PD motor dysfunction (Shen et al., 2020). As we expected, the normalized CBF values of the right putamen were correlated with UPDRS-III scores, verifying the defects of the STC circuit in PDD, consistent with previous study findings (Shen et al., 2020). A task-directed fMRI study confirmed that there were some disrupted connections in the “motor loop” connected by the SMA and putamen; these disrupted connections contributed to cognitive impairment in PD (Schonberger et al., 2015).

Compared with the PDD group, the AD group demonstrated hypoperfusion in the right IPL and right precuneus; moreover, these regions showed a positive correlation between normalized CBF values and cognitive scores. These regions are part of the default mode network (DMN) and engage in episodic memory retrieval (Lundstrom et al., 2005). Our results suggested that PDD involved more perfusion defects in motor control-related areas than AD, whereas patients with AD preferentially exhibited perfusion defects in cognitive-related areas in contrast to patients with PDD.

There were no significant differences at a rigorous ANCOVA statistical level among the three groups for absolute CBF, which was in accordance with findings by Le Heron et al. (2014). In contrast to absolute CBF values, normalized CBF was not only able to detect widespread abnormal perfusion across subcortical and cortical areas in the two dementia groups but also demonstrated divergence of the perfusion features between AD and PDD. ROC analysis showed that the normalized CBF values with significance could distinguish patients with AD from patients with PDD with relatively high accuracy.

Several limitations should be acknowledged in our study. First, due to the strict inclusion and exclusion criteria, we recruited a relatively small sample, which may affect the statistical reliability of our results, and subtler changes might be missed. Further longitudinal studies with a larger sample size are warranted to systemically analyze aberrant perfusion in patients with AD and PDD. Second, from a methodological perspective, ASL was employed here only with one PLD time, as recommended in most articles (Alsop et al., 2015). However, as ASL is a developing technique, advanced ASL procedures (multiple PLD) could provide more accurate CBF measurements. Third, although the patients with PDD suspended antiparkinsonian drugs before the scan, the long duration of antiparkinsonian medication still should be considered in our future investigation. Fourth, the value of ROC analysis based on the regions found to be different in the two disorders may be limited, a prospective validation group should be set up in future studies. Fifth, although there were no significant differences in cognitive scores (CDR and MMSE/MoCA) between PDD and AD groups in this preliminary study, the severity of dementia was not well matched among the scales, which might reduce the utility of the analyses. More detailed neuropsychological scales corresponding to cognitive domains and severity would be better for the investigation of dementia. Finally, the enrolled patients were diagnosed clinically in the present study. Multimodal imaging studies, such as those combined with specific tau and Aβ PET imaging, will be meaningful to further clarify the intrinsic mechanism of neurodegenerative diseases from multiple perspectives.

## Conclusion

In conclusion, the normalized CBF derived from ASL revealed a convergent distribution of impaired perfusion patterns shared by patients with AD and PDD. Meanwhile, AD and PDD presented divergent perfusion defection preferentially related to cognition and motor functions, respectively. Despite the overlapped perfusion distributions in both patients with dementia as compared with HC subjects, our results further suggested that there could be different pathophysiological alterations between AD and PDD in cerebral hemodynamics, enhancing the understanding of the mechanisms of dementia.

## Data availability statement

The raw data supporting the conclusions of this article will be made available by the authors, without undue reservation.

## Ethics statement

The studies involving human participants were reviewed and approved by Medical Research Ethical Committee in Clinical Medical College, Yangzhou University. The patients/participants provided their written informed consent to participate in this study.

## Author contributions

HZ and YX contributed to study concepts/study design. HC, YX, SS, and HZ performed literature research. YX, LC, XL, and WX contributed to clinical studies. HC and SS performed the statistical analysis. HC and HZ performed manuscript editing. HZ guaranteed the integrity of the entire study. All authors helped in data acquisition or data analysis and interpretation, listed have made substantial and directed contributions to the work, and approved to submit the manuscript.

## Funding

This work was supported by the National Natural Science Foundation of China (Grant No. 81471642).

## Conflict of interest

The authors declare that the research was conducted in the absence of any commercial or financial relationships that could be construed as a potential conflict of interest.

## Publisher's note

All claims expressed in this article are solely those of the authors and do not necessarily represent those of their affiliated organizations, or those of the publisher, the editors and the reviewers. Any product that may be evaluated in this article, or claim that may be made by its manufacturer, is not guaranteed or endorsed by the publisher.
